# Semantic wikis as flexible database interfaces for biomedical applications

**DOI:** 10.1038/s41598-023-27743-9

**Published:** 2023-01-19

**Authors:** Marco Falda, Manfredo Atzori, Maurizio Corbetta

**Affiliations:** 1 Neuroscience Department, University of Padova, Padova, Italy; 2 Institute of Information Systems, University of Applied Sciences Western Switzerland (HES-SO Valais), Sierre, Switzerland; 3 Padova Neuroscience Center (PNC), Clinica Neurologica, and Venetian Institute of Molecular Medicine, VIMM, Padova, Italy; 4 Department of Neurology, Radiology, Neuroscience Washington University School of Medicine, St. Louis, MO USA

**Keywords:** Software, Computer science, Software, Data publication and archiving

## Abstract

Several challenges prevent extracting knowledge from biomedical resources, including data heterogeneity and the difficulty to obtain and collaborate on data and annotations by medical doctors. Therefore, flexibility in their representation and interconnection is required; it is also essential to be able to interact easily with such data. In recent years, semantic tools have been developed: semantic wikis are collections of wiki pages that can be annotated with properties and so combine flexibility and expressiveness, two desirable aspects when modeling databases, especially in the dynamic biomedical domain. However, semantics and collaborative analysis of biomedical data is still an unsolved challenge. The aim of this work is to create a tool for easing the design and the setup of semantic databases and to give the possibility to enrich them with biostatistical applications. As a side effect, this will also make them reproducible, fostering their application by other research groups. A command-line software has been developed for creating all structures required by Semantic MediaWiki. Besides, a way to expose statistical analyses as R Shiny applications in the interface is provided, along with a facility to export Prolog predicates for reasoning with external tools. The developed software allowed to create a set of biomedical databases for the Neuroscience Department of the University of Padova in a more automated way. They can be extended with additional qualitative and statistical analyses of data, including for instance regressions, geographical distribution of diseases, and clustering. The software is released as open source-code and published under the GPL-3 license at https://github.com/mfalda/tsv2swm.

## Introduction

Despite the huge and continuous production of clinical data, their massive exploitation for data science and precision medicine remains an unsolved challenge, which semantics and collaborative approaches could contribute to solving.

Every day, thousands of clinical exams are produced and examined by medical doctors worldwide, leading to massive amounts of data produced yearly and making healthcare data one of the biggest and still unexploited digitized resources of knowledge. Medical data include many diverse data types (e.g. bio-images, bio-signals, text, genetics, and many others), which are collected with various acquisition devices and protocols making data highly heterogeneous^1–3^. The complexity and heterogeneity of biomedical datasets make it difficult to quickly get critical information about specific genes, illnesses, and treatments for precision medicine applications, as well as to perform knowledge extraction or data mining. Moreover, examining these massive datasets and combining the results from various sources is in most cases time-consuming and complicated^4^. Biomedical datasets are increasingly made publicly available leading to thousands of potentially available multi-modal data, e.g. from challenges^5–7^, open-access databases^8–12^, and scientific literature^13^. Some examples of online databases are UniProt^14^ which aims to provide comprehensive and high-quality resources on protein sequences and functional information and the Kyoto Encyclopedia of Genes and Genomes (KEGG), a professional knowledge base for the biological interpretation of large-scale molecular datasets, such as genomic and metagenomic sequences^15^.

Semantic-based approaches represent an extraordinary and increasingly exploited opportunity for biomedical sciences because they allow to create a unique, multilingual representation of medical concepts.

While in Linguistics Semantics refers to the meaning of words, phrases, or sentences, in Computer Science Semantics refers to the study of properties, categories, and relationships among concepts of a specific area^16^. Supported by formal ontologies, Semantic web technology has been adopted widely in Biomedicine for standardizing and connecting semantic datasets that are available online and accessible via the HTTP protocol^17^. For instance, the Semantic MEDLINE Database^18^ is a repository of predicates from MEDLINE titles and abstracts^19^. Percha et al.^20^ extract relations from unstructured natural language texts in biomedical literature databases. Semantic web technology is the focus of Web 3.0^21^ and the most important stack for data integration so far. It is built as a set of flexible and inter operable formats and technologies^22,23^, mostly based on the Resource Description Framework (RDF)^24^, and its query language SPARQL Protocol And RDF Query Language (SPARQL)^25^. Despite this, semantic database interfaces are still a rather difficult instrument for people who are not used to them, and raw SPARQL queries may be challenging to formulate for users without technical training; composing a good query also needs high familiarity with the dataset^26^.

Together with data and semantics, collaborative approaches represent a great opportunity for biomedical sciences, as they can allow the global advancement of biomedical data analysis. During the Web 2.0 revolution, wiki systems had wide success thanks to highly diffuse collaborative projects such as Wikipedia; wikis are websites that can be easily created and edited using a simple markdown syntax and so they are well-fitted for less technically versed people^27^. Several systems have been proposed in order to merge the best features of wiki systems and ontologies, for example Ontowiki^28^, KnowWE^29^, or Loki^30^.

One of the most complete and well-maintained systems in this context is Semantic Mediawiki (SMW)^31,32^, which is an extension of MediaWiki, the engine underlying Wikipedia. Semantic MediaWiki allows for annotating pages with semantic properties in a well-defined way and retrieving them later using simple queries; in this way, information will not get lost in weakly structured textual content. In fact, it enables wikis to present their knowledge in a computer-processable fashion.

Semantic MediaWiki has been used in several fields, for example smart cities^33^, historical studies^34^, or Biology catalogs^35^. Thanks to the completeness of the provided tools and to its ease of use, Semantic MediaWiki seems to be an ideal tool for all scenarios with highly dynamic data structures, such as biomedical research, in which multiple scopes and aims can exist in different groups, changing through the lifetime of datasets.

Semantic MediaWiki is commonly used, along with MediaWiki templates and further extensions like PageForms^36^, to generate the entities needed by each application. Currently, the set of support pages can be managed with the Page Schemata extension^37^, which stores all the needed structures in an eXtensible Markup Language (XML) format embedded in the category pages. However, this software is not easy to manage, and this is even more problematic when the number of properties increases considerably; The extension is in fact more suited to deal with just a few properties and categories.

This work proposes a tool for easing the creation of semantic databases in Semantic MediaWiki. More precisely, it consists of a command-line application to transform tabular specifications into a set of XML files ready to be imported into Semantic MediaWiki, to build the skeleton of semantic databases. There is also the possibility to fill in the prepared schema starting from data in a tabular format, which is common in products such as Microsoft Excel or OpenCalc. Note that the schema itself does not have any constraint on the properties and categories, besides those imposed by Semantic MediaWiki on the available data types, thus it can be used for modeling a wide set of scenarios. The generated schema has been empowered by providing a connection to R Shiny applications^38^ and the possibility to export Prolog predicates through MediaWiki APIs.

Semantic MediaWiki already allows importing page content from a tabular format. There are also tools able to import other formats, for example, the PageProperties extension^39^ and RDFIO extension^40^. The former easily associates semantic properties to pages without manual annotation; as far as the import procedure is concerned, it allows to map Comma Separated Values (CSV) fields to existing local properties. The latter extension is unmaintained. Using the tool described in this paper, there is also the distinctive possibility to design a Semantic MediaWiki schema starting from a tabular specification. This allows for easier interaction with end-users and makes it possible to delegate to them the design of a first draft that will be closer to their conceptualization.

R Shiny was chosen because it is commonly used for visualizing data and exposing statistical analyses on the Web. As far as semantic applications are concerned, some examples are “Semantic Scale Network (beta)”, which helps researchers and reviewers in psychological science to detect semantically related scales^41^; SemNeT, which offers researchers several tools for the analysis of their semantic network data^42^; Word Space Creator, which allows users to create semantic space models easily^43^. There are also tools for extracting knowledge from texts, like for instance the paper by Papadias, Kokle, and Tomai^44^. They are all interesting, but they are very specific applications focused on particular research needs or contexts. Our aim is instead to allow a biostatistician to develop their own data analysis software and to integrate the resulting application in Semantic MediaWiki accessing the underlying semantic data in a simple way right from R. To our knowledge, the most similar system is WINFRA, a Web-based platform for Semantic Data Retrieval and Data Analytics^45^; it seems very interesting because it integrates several analysis tools, and it should be also able to extract triples from free unstructured texts. However, it is not publicly available and therefore it was not possible to evaluate it.

As for the actual integration of R Shiny in MediaWiki, specific extensions were considered. More precisely, the extensions “R”^46^ and the extension “Shiny”^47^. Unfortunately, both are either archived or unmaintained. In the latter case, as warned in the extension site, it can be dangerous to use. Moreover, since the R code should be written directly in MediaWiki, they are cumbersome to use for a biostatistician, who traditionally works with higher level and more helpful programming environments such as, for example, RStudio. Finally, there are no indications about how to collect data from the internal Semantic MediaWiki database.

Overall, the proposed system allows to extend and query the site in a very versatile way not only as far as the visualization of collected data is concerned but also by providing a gateway for statistical, (fuzzy) logical reasoning^48,49^, and possibly predictive analyses^50^. Since the setup of such a system is very complex, a set of three Docker images orchestrated by Compose has been provided.

## Implementation/methods

### Semantic MediaWiki foundations

The fundamental units of Semantic MediaWiki are the pages, as usual in any wiki. Being an extension of MediaWiki, the language for formatting text is the wikitext markup^51^ and it is possible to categorize pages for easier organization. The innovative point of Semantic MediaWiki is that pages can also be annotated with properties associated with a type, and this allows for using them almost automatically for various operations on data; in this sense, they can be defined as “semantic properties”, in addition to the more traditional meaning related to the machine-processability of information and exchange between agents^52,53^.

The most common use of Semantic MediaWiki is to structure a wiki as a database, and to this aim, the MediaWiki templates are essential since they ensure uniformity in annotations; they can be seen as lexical transformations like C macros being the output of the preprocessor still a (wiki)text^54^. Through templates, it is also possible to associate categories to pages, which correspond to Ontology Web Language (OWL) classes^55^. These are very useful not only for organizing content but also for subsequent queries and can be thought of as tables in relational terms. Categories are also the link between pages and the forms provided by the PageForms extension^36^. Thanks to this extension it is indeed possible to generate a template for the pages of a given category, and obtain in this way an association between the properties in the template and the input controls in the forms.

Once data have been modeled as page properties, it is possible to consult the site through special associated pages or with a multifaceted search interface provided by the Semantic Drilldown extension^56^. A more flexible method is to use simple queries which have a syntax similar to the categories and properties annotations and use the so-called “printouts” to define projections, that is variables prefixed with a ‘?’, like in SPARQL. These queries are known as “ask queries”. For example to retrieve all patients with a temperature greater than 37$^{\circ }$ and report their temperature the following query can be specified:
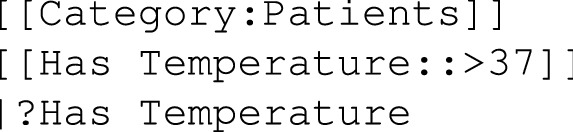


Note that there is a reference category, which is used to select pages and their properties.

Wikis are not Content Management System (CMS) and therefore they are not designed to manage user access rights. *Ad hoc* extensions have been created, for example, the SemanticACLs extension^57^ to emulate such functionalities by assigning roles to users or groups, or the Lockdown extension, which can protect special pages^58^.

Query results are typically presented as tables, however, there are other possible representations, thanks to the semantic nature of properties. The Semantic Results extension^59^ provides several additional formats, while the Maps extension^60^ allows for placing entities on geographical maps using the coordinates associated with a property of them; also charts can be traced on query results.

### Semantic MediaWiki enhancements

**Figure 1:**
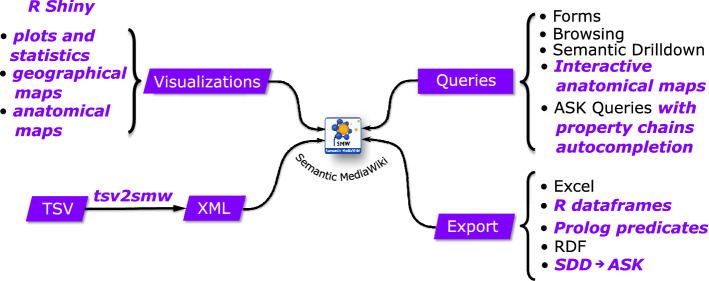
**SMW aspects that have been extended:** several SMW aspects have been extended and they have been written in purple. Their parent groups have been represented by parallelograms when related to inputs, otherwise they have been represented by rectangles [Created with Inkscape 1.1.2 (b8e25be833, 2022-02-05) by the first author; https://inkscape.org/].

Several aspects of Semantic MediaWiki were addressed and extended: semantic properties for annotating wiki pages and querying them, format facilities to display results, and the command-line tool for generating pages with the previous features starting from a TSV file. In Fig. 1, they have been highlighted in purple.

#### Database querying

**Figure 2:**
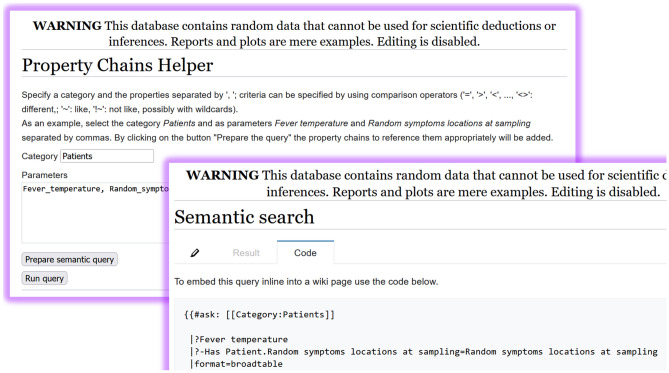
**Property chains auto-completion:** the user selects the category and the list of properties and then a semantic query can be generated complete with the correct chains, possibly inverse [Screenshots from the public demo site (see Data availability section) taken by the first author].

As far as queries are concerned, the multifaceted search interface provided by the Semantic Drilldown extension (SDD)^56^ has been enriched with groups of properties and the possibility to save selections as ASK queries (*vide supra*). When properties belonging to pages with different categories are to be retrieved together, there must be a connection property, and property chains must be provided. Expanding on the previous example, suppose that there is a “Has Patient” relation between Patients and Samplings, then all patients with a temperature greater than 37$^{\circ }$ that have a positive sampling can be retrieved, and temperature and sampling reported; observe that a property chain must be used also in the printout and that may be also inverse (minus sign prefixed):
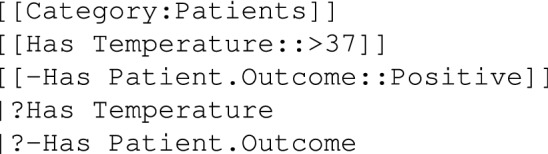


To help with property chains, a custom extension has been developed to auto-complete them^61^. When writing a query, it is sufficient to specify the category and the list of its properties, possibly associated with a filter (Fig. 2).

#### User management

Five groups of users have been envisioned: *sysops* have read/write permissions to all pages and administrative privileges; *editors* have read/write permissions to data pages and possibly a few other auxiliary pages; *viewers* have read-only permissions to data pages and possibly to a few other auxiliary pages; *exporters* can only export data from special pages; *guests* can just write and read data pages but not query them. The SDD extension is aware of the group and it allows for exporting results only to the users belonging to the *exporters’* group. Also, the general export facility and the ask queries are available exclusively to these users. Notice that user groups are not mutually exclusive, for example, a viewer could also export.

#### Data exploration

A more versatile way to extend representations has been developed: a link with R scripts and Shiny web apps. It was built using widgets, a sort of JavaScript modules in MediaWiki. R Shiny applications can query the Semantic MediaWiki site using MediaWiki APIs, and for easing this task auxiliary R functions have been implemented for retrieving all properties or data by composing a well-formed query starting from its basic components. The code is in the Github repository at https://github.com/mfalda/tsv2swm and in Appendix S1, Source Code S1.1. Additional overlays for representing anatomical maps have been created using pyramidal tiles^62^.

#### Schemata design and creation

Medical doctors very often collect data in Microsoft Excel and, in general, the tabular format is also quite versatile for specifying a schema and even for sharing it. Semantic MediaWiki can import XML documents, but this is a format that has been designed to be easily parseable by machines while for (non-trained) humans it is difficult and very redundant: the Tab Separated Values (TSV), which mimics a simple table, is a more straightforward format. For this reason, a command line software for transforming from a conveniently crafted TSV file to Semantic MediaWiki XML has been developed. The grammar of the tabular input schemata is reported in Supplementary, Source Code S2.1, in the usual Backus-Naur form^63^ and, as it can be seen, most basic SMW types and constructs have been taken into account.

Three main functionalities have been implemented:

1. Generate the schema from a semantically enriched TSV format. The TSV has been enriched (semantically) in order to express several features of Semantic MediaWiki such as categories and their hierarchies, auxiliary pages, domains of categorical fields, numerical ranges, and so on.
2. Translate a TSV table into an XML file ready to be imported into Semantic MediaWiki.
3. Generate a TSV table or an XML data file filled according to the constraints in point 1.

The command-line tool has been developed in C# (.Net Core); more details are reported in Supplementary Materials, Appendix S3. C# is a programming language that has the speed of development typical of dynamic languages like Python and Perl and the rigor of strongly typed languages such as Java and C++. It also provides convenient functional constructs in the form of Language-INtegrated Query (LINQ) expressions though it does not have the powerful type system of true functional languages like Scala or Haskell^64^.

### Qualitative survey on usability and relevance

To obtain qualitative feedback from colleagues, a short survey was submitted to different clinical units within the department. It was composed of five questions based on a scale from 1 (easy) to 5 (hard) plus a neutral position (“Don’t remember”):

1. *How easy is data exploration?*
2. *How quick is data entry?*
3. *How intuitive is the interface?*
4. *How useful are statistical graphs?*
5. *How appropriate is data export?*

Additional free text fields were provided for general comments and notes.

## Results

### Semantic databases creation

The results of this work include both the automatic generation of building blocks starting from TSV schemata and data and the extension of the basic representations using R Shiny applications. This eased the creation of several database schemata containing tens or hundreds of properties. The work in this paper was developed for providing a set of databases at the Neuroscience Department, University of Padova. Their schemata have been proposed to several research groups: 12 databases have been prepared for very diverse research fields such as Neurology, Otorhinolaryngology (ORL), or Psychiatry (Table 1). More are planned.

**Table 1:** **Implemented database schemata:** list of the currently implemented database schemata at the Neuroscience Department, University of Padova.

Unit	Topic	Properties per patient	Properties per visit
Neurology	ALS	105	63
	Movement disorders	34	136
	Neurodegenerative diseases	30	0
	Neuro-oncology	40	40
	Stroke	59	78
ORL	Audiological prostheses	194	25
	Head-neck tumors	109	0
Psychiatry	Eating disorders	116	0
Physical medicine	Lymphedema	67	30
	Osteoarthrosis	33	55
	Post COVID	32	29
	Scoliosis	31	104
TEST	COVID cases	31	10

As a reference and complete example, a database inspired by the COVID-19 virus study in^65^ has been created using their public data. Additional fields filled with random data were added in order to show all the features provided by the software, such as geographical locations. All plots and maps in the following have been built from these data.

The Virus site was obtained starting from the TSV files in the Github repository. First, the structure was created using the command
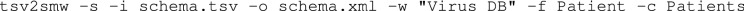


the options are explained in the Supplementary Materials, Appendix S3. The previous command created an XML file ready to be imported into Semantic MediaWiki.

The XML with the citizen data was prepared with the command
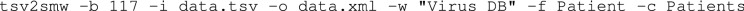


**Figure 3:**
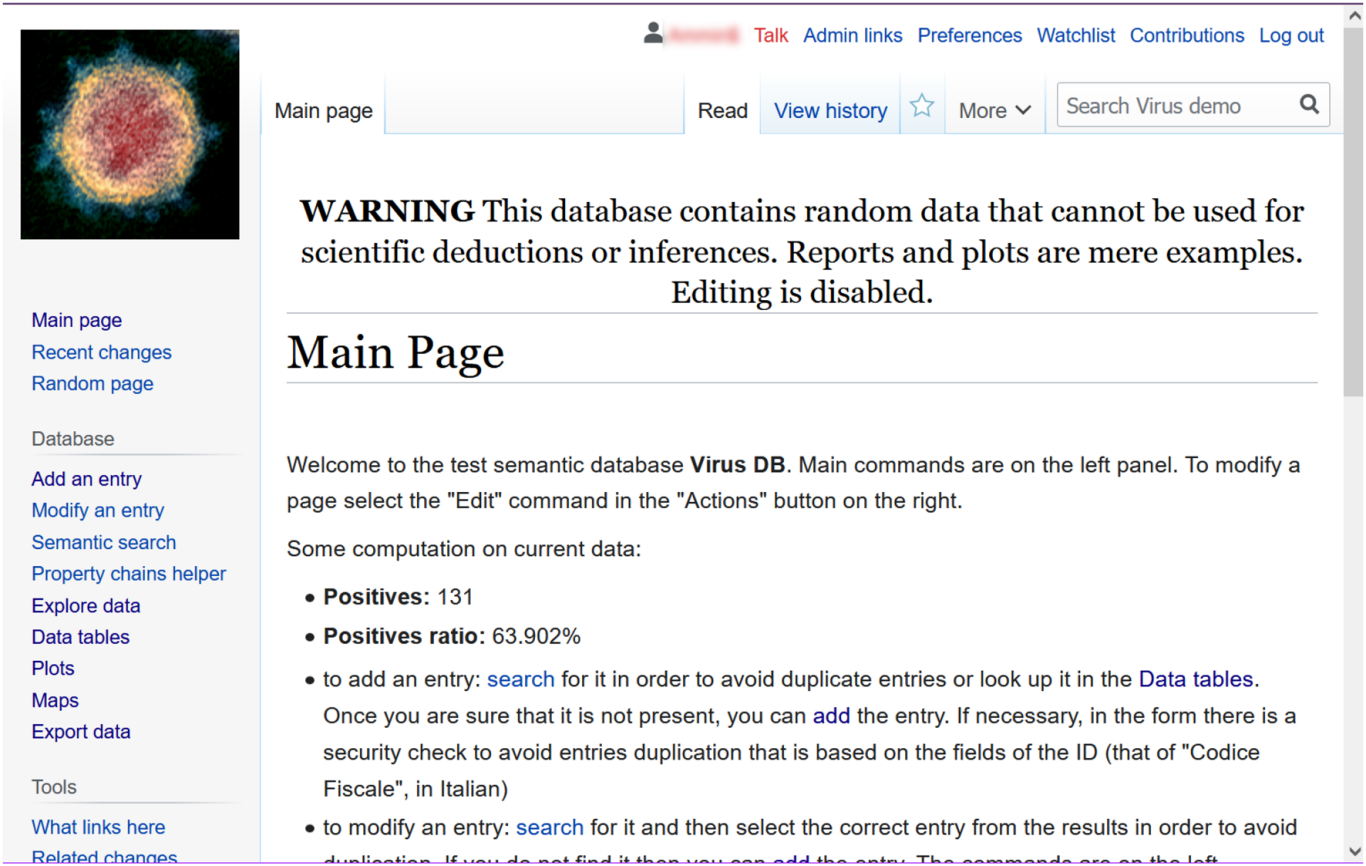
**The main page of a typical database:** the typical homepage for a generated site in which main operations are explained. It can be customized with a template in tsv2smw or simply by editing its underlying markdown representation. [Screenshots from the public demo site (see Data availability section) taken by the first author].

The home page, shown in Fig. 3, contains a small help guide with links to the pages for populating and exploring the database which are grouped into three areas: database building and modification, query, and statistics, and export. The same links are also present in the sidebar on the left.

### Database querying and data exploration

Ternary predicates representing subject, property, and object are generated ready to be easily imported into a Prolog interpreter, and then queried. For example, the following query gets all the patients with a temperature greater than 37$^{\circ }$:
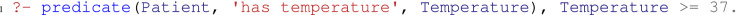


An ad hoc pack^66^ that provides predicates for logging into Semantic MediaWiki, loading predicates, and querying them directly from SWI-Prolog has been developed. The synopsis of the main $ask\_query/8$ predicate is:
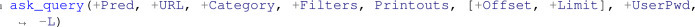


Complex properties, which are typical of medical domains, can be enriched with additional information. For example, in the Audiology schema properties range from 1 to 11 words (in Italian, acronyms are used and some invariable words omitted, as usual), the longest being the following one, translated into English:

“Pure tone average of vocal gain during last control exam with an intracochlear device on left ear.”

**Figure 4:**
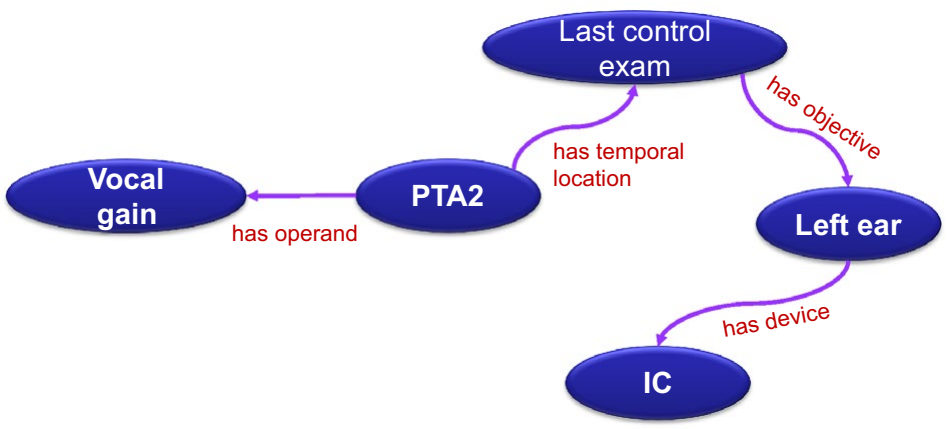
**Modeling of a complex property:** the possible logical decomposition of the complex property “Pure tone average of vocal gain during last control exam with intracochlear device on left ear”. Conceptually, an unstructured text representing the property in the database would expand to a set of logical clauses that can be queried and summarized in Prolog [Created with Inkscape 1.1.2 (b8e25be833, 2022-02-05) by the first author; https://inkscape.org/].

The previous complex property can be modeled as in Fig. 4 and vertices can be embedded in the property page itself using custom relations, such as those indicated in the figure.

Using such modeling, the exported Prolog predicates would be like the following:
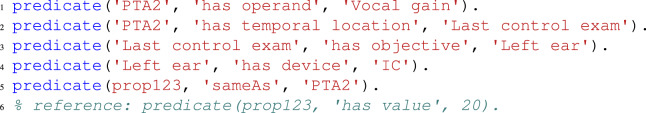
 which could be queried for example as
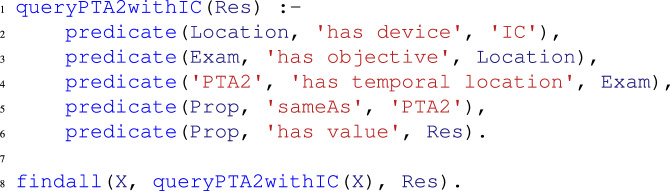
 Obviously, some of the previous clauses could be also omitted to obtain wider result sets.

The R data frame format is perhaps less useful, as it could also be obtained using the *read.table* function in R starting from a CSV file already exportable from Semantic MediaWiki, or from the Excel export format using the functions in the *readxl* package, possibly specifying additional import options. As said before, the first idea is used also for the embedded Shiny web applications.

#### Statistical applications

**Figure 5:**
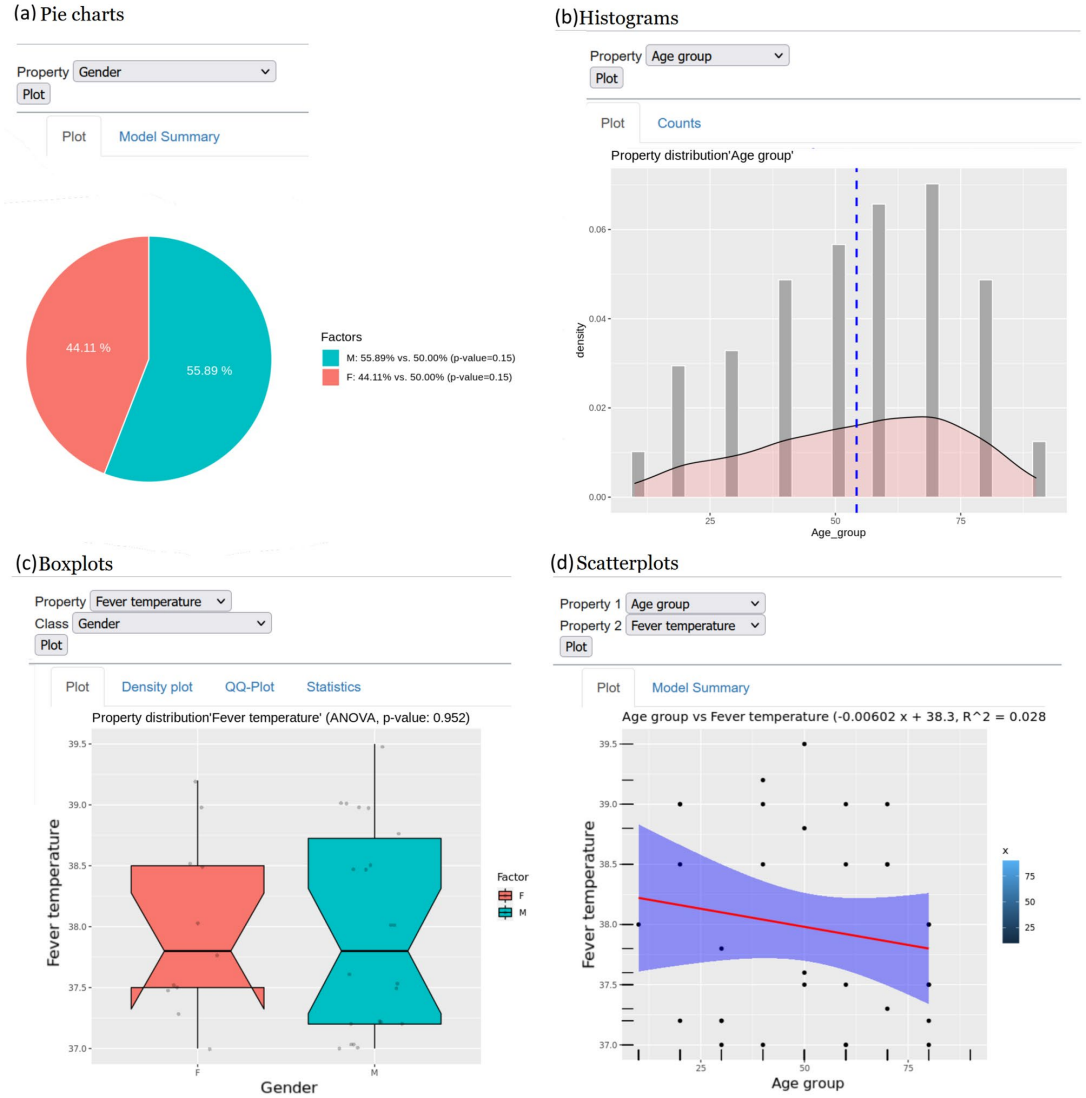
**Examples of embedded Shiny applications:** (**a**) pie charts with proportion tests, (**b**) histograms, (**c**) boxplots with ANOVA, (**d**) scatter plots with regression [Screenshots from the public demo site (see Data availability section) taken by the first author].

To demonstrate the benefits of connecting to R Shiny, some descriptive statistics sample applications and tests on bivariate public or random data have been created, ready to be further developed; they are already apt to get an idea about data distribution and relations. In particular, there are pie and bar graphs with associated p values obtained from proportion tests adjusted with Benjamini–Hochberg False Discovery Rate (FDR) (Fig. 5a), histograms (Fig. 5b), box plots (Fig. 5c), and scatter plots (Fig. 5d). As an example, graphs could be partitioned with facets: it all depends on the underlying R code, which will certainly be easy for an average biostatistician. Applications can also become more complex, for example in the box plots application associated analyses depending on the underlying distributions have been added; in the scatter plot application linear regression fitting.

**Figure 6:**
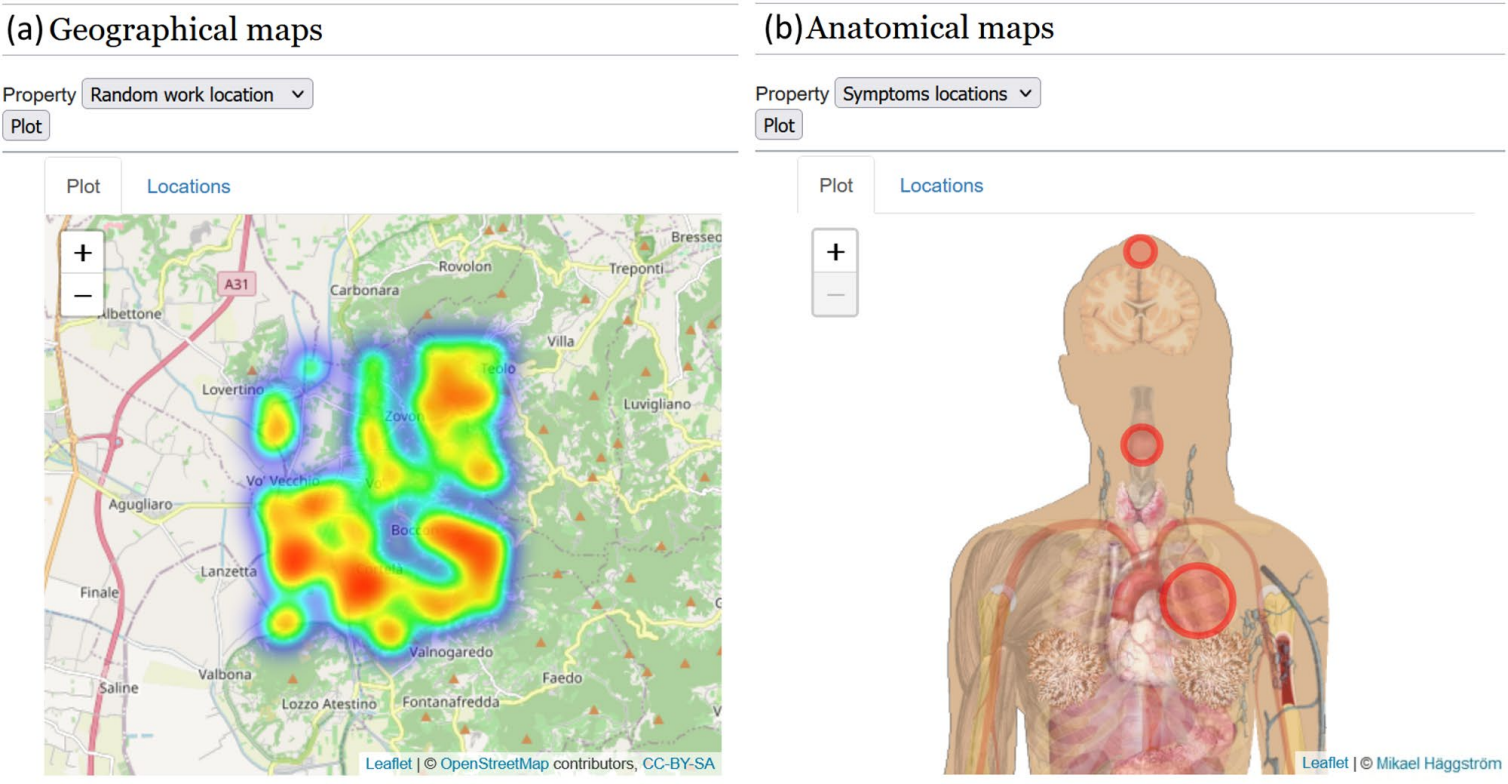
**Two examples of maps:** (**a**) geographic map with an overlaid heatmap. (**b**) Anatomic map in which placeholders’ radii are proportional to the cardinality of the patients’ sets with specific symptoms [Screenshots from the public demo site (see Data availability section) taken by the first author. The anatomical map in Fig. 6b is from Wikimedia Commons; the file is made available under the Creative Commons CC0 1.0 Universal Public Domain Dedication at https://commons.wikimedia.org/wiki/File:Female_shadow_with_organs.png].

#### Maps

Maps have been added both using the Map extension and by developing an R application. In the latter case, more advanced features can be implemented. As an example, in the case of anatomical maps, placeholders’ radii have been related to the cardinality of the set of patients having a particular random symptom (Fig. 6b), while a heatmap has been used to represent the density of random locations in geographic maps (Fig. 6a). The possible data analyses and representations are only limited by the underlying R framework.

#### Answers to the qualitative survey

Out of the 8 sub-units for which the 12 implemented databases were developed (Table 1), 5 replied. The results were encouraging, having an average score of $4.3 \pm 0.48$ over a maximum of 5. More details have been reported in Supplementary Materials, Appendix S5.

The first question about the ease of data exploration obtained an average score of $4.4 \pm 0.5$ over 5. Question 2 on the speed of entry received a low vote scoring an average of $3.4 \pm 1.6$ over 5. Question 3 is about the user interface and received an average score of $4.2 \pm 0.8$. Question 4 refers to plots and statistical analyses, and received a score of $4.3 \pm 0.5$, however, it presents a slice of unexpressed opinions. The last question obtained the lowest number of answers and scored an average of $4.7 \pm 0.6$.

## Discussion

This work presents a first, complete tool for designing a Semantic MediaWiki instance to be used as a semantic database enriched with statistical analyses. The choice of R Shiny as the underlying engine for computations permits extending it with a wide range of embedded interactive applications.

In fact, this software should allow for a more straightforward and direct design process and foster a potentially widespread adoption of such a flexible semantic platform as Semantic MediaWiki in all those data modeling scenarios in which one or more “one-to-many relations” are present in the design due to functional dependencies in the data themselves. In the case annotations can be linked to known formal (bio)-ontologies, special semantic properties can be used to establish equivalences translated into owl:SameAs statements, and in this way different datasets can be connected and collaboration on inter-specialty data becomes possible, possibly leading to a globally “Medical scientific wiki database”.

The database built by importing the XML file generated by tsv2smw is already using simple forms for entering data based on the PageForms extension. The only attention was to provide a uniqueness check, since the *unique* constraint in Semantic MediaWiki merely signals conflicts but does not prevent them. It was indeed decided to name the entities with progressive numbers, in order to implement a more general abstraction. In fact, when personal details are modeled it would perhaps be simpler to use personal data for identifying them uniquely. However, there is a MediaWiki facility named DISPLAYTITLE^67^ which allows for changing dynamically the titles of the pages therefore this should not be a problem.

Another interesting point that has been addressed is the explicit representation of missing data: besides the usual classification^68^, a field could be without a value because it has not yet been considered, therefore some Boolean properties have been extended to a “three-valued” logic that allows for a third possibility which stands for “not available” (*“tertium datur”*).

Currently, the system is hosted in a closed intranet behind perimeter firewalls, and in exported data, sensitive information is omitted. Should privacy concerns arise, a symmetric deterministic client-side encryption^69^ will be enforced on certain sensitive fields that are not useful for downstream processing. An important point will be the treatment of geographical coordinates since they are sensitive but useful for geographic clustering; a possible solution will be to aggregate locations until a certain population threshold will be reached, in order to make individuals identification very hard. More pervasive data alterations should be limited since analyses on encrypted data would be more complex to manage^50^. Two-factor authentication is available to users.

Prolog predicates could be more familiar and simpler than SPARQL syntax for some users and this language is already adopted in some systems such as AllegroGraph^70^ or Loki^30^; its syntax is not difficult, being based on facts, rules, and goals^71^. To be able to operate directly from the site, a special page based on Tau-Prolog^72^, an open-source Prolog interpreter written in JavaScript, could be included in order to perform limited-depth queries. Alternatively, a SWISH embedded site^73^ could be added. In both cases, a set of predefined queries could be provided according to the underlying properties and types.

The possibility of interfacing R scripts is ideal for better decoupling the development of (intelligent) analysis modules by people with more specialized skills: a biostatistician could comfortably work in his own development environment and then transfer the application she created to the developer in order to integrate it in the site.

Also, Anatomical maps can be created using R and they can be used to trace, for example, the successive locations of tumor relapses in individual patients or, given a location, all patients with that particular symptom (Fig. 6b). The advantage of a “symbolic” approach is that a first level of abstraction is already introduced, which could be further exploited through hierarchical clustering or clustering on categories. All this expressive power comes precisely from the possibility to use R, or also another data analysis framework, for dealing with data.

Some limitations emerged and will be addressed. First, the flexibility of the underlying TSV schema should be enhanced, since there persist some peculiarities typical to the context in which the system is currently used, namely the core patient-visit relation at the base of all the currently developed databases. This flexibility requires careful parsing of the feeble structure equipping the TSV input files. It could be also useful to give the possibility to operate on differential TSV schemata in order to make maintenance easier and allow for simpler amendments.

Another critical aspect concerns the possibility of sharing common categories in a wiki-farm configuration. To segregate relevant data to different research groups, a more robust and secure way is to set up a set of wikis; an alternative would be to configure a single site using namespaces. In this case, a triple-store could be a viable solution, and greater integration of Semantic MediaWiki with Wikidata or SPARQL endpoints , in general, would be ideal.

A system validation among users demonstrated the usability of the developed Semantic MediaWiki interface by bio-medical users and it suggested improvements for future interface development. There were no major negative comments by collaborators related to data exploration (Question 1). According to users’ answers, semantic properties and data exploration are topics of interest and could benefit biomedical researchers’ work. Data entry is time-consuming, which may explain the relatively low scores (Question 2). The input controls are in standard HTML, and they cannot be made easier. Question 3 is about the “intuitiveness” of the system. The relatively low scores reflect, in our opinion, the organization of the menu commands. This aspect could be improved by instrumenting the graphical interface and timing the users’ mouse actions. Question 4 was about statistical graphs and received relatively few answers, possibly because the end-users are medical doctors who are not as familiar with statistics. Those who voted indicated high interest, revealing the importance of a tool for statistically plotting the results. The last question was about data export, and it received few answers. This result might be due to the relatively small amount of stored data. Also, biostatisticians use this function more commonly than medical doctors we used to test the survey. Data export can improve by making the format similar to the more familiar Excel sheets.

## Conclusion

Semantic databases built with wiki platforms are flexible and easy for end-users even in the case of complex biomedical data, thanks to the possibility to extend them and to customize the interface, however, they must be created manually or through embedded forms, and therefore it is difficult to create and manage large sets of properties with them.

In this paper, a tool for creating Semantic MediaWiki XML schemata from specifications in a simple TSV format has been proposed along with a method for embedding statistical applications in R Shiny and for exporting Prolog predicates. This allows for increasing the expressiveness of the platform by producing plots, statistical analyses, and rich maps. Additional embedded applications will be studied for other data types, for example, survival curves for time series, Gaussian mixture models for spatial clustering, and Natural Language Processing algorithms for medical reports.

## Supplementary Information

Supplementary Information.

## Data Availability

A demonstration of the system containing public data adapted from a study about COVID-19^65^ with additional random data is available online at https://dbnsdemo.neuroscienze.unipd.it; access can be allowed by the corresponding author on reasonable request.

## Code availability

Since the setup process involving Semantic MediaWiki and its required extensions, the R Shiny server, and an underlying relational database management system is very complex, a set of Docker images orchestrated by Compose has been created. All code has been published as open-source with GNU Public License v3 and it is available at https://github.com/mfalda/tsv2swm and https://github.com/mfalda/docker-smw (Supplementary materials, Appendix S4).
