# Supplementary material for: Semantic wikis as flexible database interfaces for biomedical applications
Source: Sci Rep. 2023 Jan 19;13:1095. doi: 10.1038/s41598-023-27743-9 (PMC9851594; doi:10.1038/s41598-023-27743-9)
Supplement: Supplementary file 1 — Supplementary Information. [file 41598_2023_27743_MOESM1_ESM.pdf]

Supplementary materials for the paper "Semantic wikis as flexible database interfaces for biomedical applications"

Marco Falda[1,*], **Manfredo Atzori**[1,2], **and Maurizio Corbetta**[1,3,4]

[1]Neuroscience Department, University of Padova, Padova, Italy
[2]Institute of Information Systems, University of Applied Sciences and Arts of Western Switzerland (HES-SO Valais-Wallis), Sierre, Switzerland
[3]Padova Neuroscience Center (PNC), Clinica Neurologica, and Venetian Institute of Molecular Medicine, VIMM, Padova, Italy
[4]Department of Neurology, Radiology, Neuroscience Washington University School of Medicine, St.Louis, MO, USA
[*]Corresponding author: marco.falda@unipd.it

Appendix S1 - R code for querying Semantic MediaWiki

Semantic MediaWiki provides an extension named Semantic Result Formats for displaying results as tables, plots, or other kinds of visualizations. There is also the Map extension for geographic maps. The limit of these facilities is that they must be extended in PHP and they are tightly related to the internals of MediaWiki and Semantic MediaWiki. Maps extension for example was born as an extension of Semantic MediaWiki and indeed it was named Semantic Maps; now, while still benefiting from semantics, it has a more general application context (see https://maps.extension.wiki/wiki/Maps_for_MediaWiki). For this reasons, a set of R functions has been developed; they are reported in Source Code S1.1. These functions can be invoked inside R Shiny applications to get data from Semantic MediaWiki. In turn, these applications which are served on default port 3838 can be embedded using Widgets (https://www.mediawiki.org/wiki/Extension:Widgets).

```
require(WikipediR)
library(rjson)


get_all_properties <- function(host, wiki, limit=5000)
{
        logged.in <- login(paste(host, wiki,
         ↪  'api.php?action=ask&query=&format=json',
                                                    sep='/', 'User', 'Password')
        stopifnot(logged.in)

        url <-
         ↪  sprintf("%s/%s/api.php?action=browsebyproperty&limit=%d&format=json",
                                 host, wiki, limit)

        response <- httr::GET(url)

        httr::stop_for_status(response)
        response_text <- httr::content(x = response, as = "text")

        result <- fromJSON(response_text)

        props <- lapply(result$query, function(x) x$label)

        return(props)
}

```

```r
query_smw_TSV <- function(host, wiki, category, printouts, limit=5000, offset=0, verbose=F)
{
        logged.in <- login(paste(host, wiki, 'api.php?action=ask&query=&format=json',
        sep='/'), 'User', 'Password')
        stopifnot(logged.in)

        category <- URLencode(category)
        printouts <- URLencode(paste(printouts, collapse='/?'), reserved=F)
        printouts <- gsub("?", "-3F", printouts, fixed=T)
        url <- sprintf(paste0("%s/%s/index.php?title=Special:Ask&x=-5B-5BCategory:%s-5D-5D",
                                        "/-3F%s/format=csv/offset=%d/limit=%d"),
        host, wiki, category, printouts, offset, limit)
        if (verbose) {
                cat(url, "\n")
        }
        response <- httr::GET(url)

        httr::stop_for_status(response)
        response_text <- httr::content(x = response, as = "text")

        return(read.csv(text=response_text))
}
```

Source Code S1.1. Code for retrieving data from Semantic MediaWiki in R.

Appendix S2 - Backus-Naur grammar for the TSV schemata

The TSV files containing the schemata of the database have been enriched by allowing to specify diverse features of a Semantic MediaWiki site. Namely, the following aspects have been addressed:

- categories;
- types and input controls for properties;
- groups of properties;
- super-properties;
- sub-pages;
- embedded templates;
- information tooltips;

The BNF grammar is reported in Source Code S2.1 .

```
<start> ::= <Category part>* <Subpage part>* <Main part>

<Category part> ::= <Category label> <Category body>+ <Emptyline>

<Main part> ::= <Section label> <Section header> <Section body>+ <Emptyline>

<Subpage part> ::= <Subpage label> <Section header> <Section body>+ <Emptyline>

<Emptyline> ::= \n\n

<Category label> ::= Category:<Name>\n

<Section label> ::= Section:<Name>\n

<Subpage label> ::= Subpage:<Name>/<Category>\n

<Name> ::= [A-Za-z0-9_]+

<Category> ::= <Name>

<Category body> ::= <Name>\n

<Section header> ::= Group<TAB>Super-property<TAB>Property
<TAB>Type<TAB>Domain<TAB>Option<TAB>Info\n

<Section body> ::= <Name><TAB><Name><TAB><Name<TAB><Type>
<TAB><Domain><TAB><Option><TAB>.*\n

<TAB> ::= \t

<Type> ::= Text|Date|Number|Geographic coordinates|List|File|Subpage|Boolean

<Domain> ::= (<Name>,(<Name>,)+)|<Subpage label>|<ParserFunction>

<ParserFunction> ::= /* A MediaWiki parser function (see text) */

<Options> ::= Integer|Extended|Exclusive|Computed|Repeated
```

Source Code S2.1. extended TSV format in Backus-Naur form.

Appendix S3 - The `tsv2smw` tool

`tsv2smw` is a command line application developed for the .NET Core platform and available at https://github.com/mfalda/tsv2swm It can be compiled and run in all supported Operating Systems (Table S3.1). The command line options are in Figure S3.1.

TSV2SMW1.0.0-beta

–schema Process the schema file.
–random-sizes Set the number of pages.
–random-TSV Create a random TSV.
–begin-ID Set the initial ID.
–input Required. The input file
–language Interface language.
–output The output XML file.
–wiki Required. The name of the wiki.
–cat-name The name of the main category.
–users-file The TSV file with the user IDs.
–ft-name Main form and template names.
–help Display this help screen.
–version Display version information.

Figure S3.1. `tsv2smw` command options.

Table S3.1. .NET 6 - Supported OS versions adapted from https://github.com/dotnet/core/blob/main/release-notes/6.0/supported-os.md.

**Windows**	
**OS**	**Version**
Windows Client	7 SP1[1], 8.1
Windows 10 Client	Version 1607+
Windows 11	Version 22000+
Windows Server	2012+
Windows Server Core	2012+
Nano Server	Version 1809+
**Linux**	
**OS**	**Version**
Alpine Linux	3.12+
CentOS	7+
Debian	10+
Fedora	33+
openSUSE	15+
Red Hat Enterprise Linux	7+
SUSE Enterprise Linux (SLES)	
Ubuntu	16.04, 18.04, 20.04+
**macOS**	
OS	**Version**
macOS	10.14+
**Android**	
**OS**	**Version**
Android	API 21+

In this application, the XML files are generated by adding pages (<*page*> tag) after a <*siteinfo*> section which defines

namespaces. Such pages are based on models represented by a fixed set of XML files, listed in Table S3.2.

Table S3.2. List of the built-in templates for generating pages in XML files.

File	MIME Type	Use
category.xml	text/x-wiki	Category pages
css_page.xml	text/css	CSS pages in MediaWiki namespace
form.xml	text/x-wiki	Form pages
instance.xml	text/x-wiki	Pages with data
js_page.xml	text/javascript	JavaScript pages in MediaWiki namespace
property.xml	text/x-wiki	Property pages
raw_page.xml	text/x-wiki	Simple pages
simple_form.xml	text/x-wiki	Forms for repeated sections (used in embedded templates)
simple_template.xml	text/x-wiki	Embedded templates
site_info.xml	text/x-wiki	Initial namespaces definitions
template.xml	text/x-wiki	Template pages

Other auxiliary pages can be inserted and they are automatically associated with a MIME type according to the namespace specified in an initial comment in these files. They allow for two parameters: The main category and the main template names. Two (or more) sub-directories are used to localize them in case the predefined parameters are not sufficient. The predefined set of such files is reported in Table S3.3.

Table S3.3. List of the predefined set of auxiliary files ready to be inserted in the site skeleton.

File	Mandatory	Description
bibliography	NO	example of SemanticCite bibliography
common.css	**YES**	the common styles
(enlit)/common_param.js	**YES**	the common JavaScript functions
(enlit)/forced_globalnotice.md	NO	a global notice: useful for prototypes
(enlit)/form_SearchEntry_param.md	**YES**	the form for searching entries
(enlit)/introduction_param.md	**YES**	text for the main page
(enlit)/modify_entry_param.md	**YES**	advice for editing data pages
(enlit)/pivot.js	NO	scripts related to Pivot skin
(enlit)/plots.md	**YES**	the page with plots
(enlit)/sidebar_param.md	**YES**	the sidebar configuration
(enlit)/template_SearchEntry_param.md	**YES**	the template for searching entries
(enlit)/widgets_Timeline_param.md	**YES**	the page for timelines
gadgets_definition.md	NO	page with gadgets definitions
gadgets_encdec.js	NO	example of gadget
pivot.css	**YES**	styles related to Pivot skin
smw_import_ncit.md	NO	example of NCIT ontology
Smw_import_vcard.md	NO	example of Vcard ontology
template_SciteAPAJournalResourceFormatter.md	NO	example of bibliographic format
widgets_Iframe.md	**YES**	used by ShinyPlotSrv page
widgets_ShinyPlotSrv.md	**YES**	used for linking with R Shiny apps

Appendix S4 - Installing the Docker images

The setup of a SemanticMediaWiki site is a complex and time-consuming process. Moreover, the command line tool `tsv2smw` generates XML pages that use several additional extensions, and it also relies on R Shiny server in order to display plots and statistical analyses. For these reasons, a set of three Docker images coordinated by Docker Composer have been prepared. They can be found at https://github.com/mfalda/docker-smw.

S4.1 Bot credentials

Create a file named **credentials.R** in the *shiny/shiny-server* directory containing the user and password variables, for example:

```
username <- 'ForAPI'
password <- 'example'
```

S4.2 Database parameters

Fix the username and password for the database in the YAML file; this will be required in MediaWiki setup. Take note also of the database server name if you change it.

S4.3 Start the containers

For starting this set of containers you must use Docker Compose (please check that for the first invocation the line for copying the file **LocalSettings.php** is commented; this directive should be located at line 20 of the YAML file):

```
$ sudo docker-compose up
```

Then, access it via http://localhost:8081/mw-config/ in a browser.

S4.4 MediaWiki configuration

When accessing the web server for the first time, a brief setup process will be started. Please remember to change the Database host to **database**. In order to enforce user roles you have to opt for a private wiki. For security reasons, there is not an email server in the Docker images.

Once done,

1. save the LocalSettings.php file in the *wiki* sub-directory,
2. uncomment the relevant line in the YAML file (should be line 20),
3. append at the end of the file the contents of the 'LocalSettings.local.php' file,
4. and finally restart the images.

S4.5 Access the web server

Before accessing the web server at http://localhost:8081/ update it with the command

```
sudo docker-compose exec smw php maintenance/update.php --quick
```

S4.6 Completing bot setup

Please create a user for accessing APIs having the previous credentials set for **ForAPI** (from the Create user special page) and give it the **bot**, **view**, and **export** privileges (from the User rights special page).

S4.7 Import data

Finally, import the XML files with *maintenance/importDump.php* and update as usual:

```
$ sudo docker-compose exec smw php maintenance/importDump.php schema_Virus.xml
$ sudo docker-compose exec smw php maintenance/importDump.php data_Virus_pos.xml
$ sudo docker-compose exec smw php
↪ extensions/SemanticMediaWiki/maintenance/rebuildData.php
$ sudo docker-compose exec smw php maintenance/update.php --quick
$ sudo docker-compose exec smw php maintenance/runJobs.php
```

Please ensure that the job queue is empty.

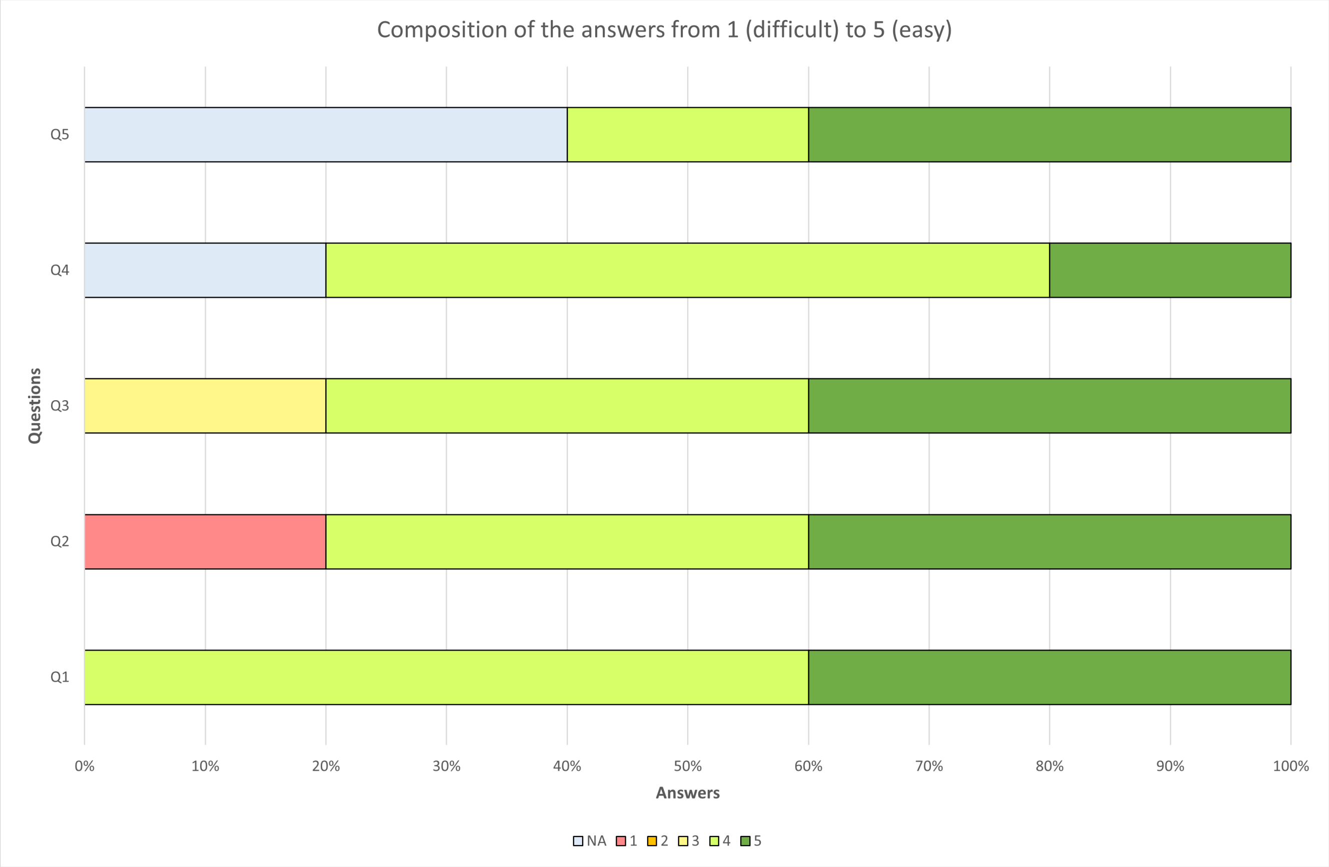

Figure S5.1. Answers to the survey: overall chart of the answers to the five questions proposed to our colleagues about the system.

Appendix S5 - Qualitative survey about the system

To obtain qualitative feedback from colleagues, a short survey was submitted to different clinical units within the department. It was composed of 5 questions based on a scale from 1 (easy) to 5 (hard) plus a neutral position ("Don't remember") that has been omitted from the averaged scores:

1. How easy is data exploration on a scale from 1 (complex) to 5 (easy)?
2. How quick is data entry on a scale from 1 (slow) to 5 (quick)?
3. How intuitive is the interface on a scale from 1 (not at all) to 5 (very intuitive)?
4. How useful are statistical graphs on a scale from 1 (not at all) to 5 (very helpful)?
5. How appropriate is data export on a scale from 1 (not at all) to 5 (entirely)?

Additional free text fields were provided for general comments and notes.

Out of the 8 sub-units for which the 12 implemented databases were developed, 5 replied. The results were encouraging, having an average score of 4.3 ± 0.48 over 5 (Figure S5.1).

S5.1 Question 1

The first question asked to the users in the system validation questionnaire was related to the ease of data exploration (Figure S5.2). This question obtained an average score of 4.4 ± 0.5 over 5, demonstrating that the system is deemed easy to use by most collaborators. There were no major negative comments by collaborators related to data exploration and the main ones were minor ones related to concrete usage options, which could be easily improved with a slightly longer explanatory introduction. According to users' answers, semantic properties and data exploration to them seem a topic of strong interest and it could benefit the work of biomedical researchers.

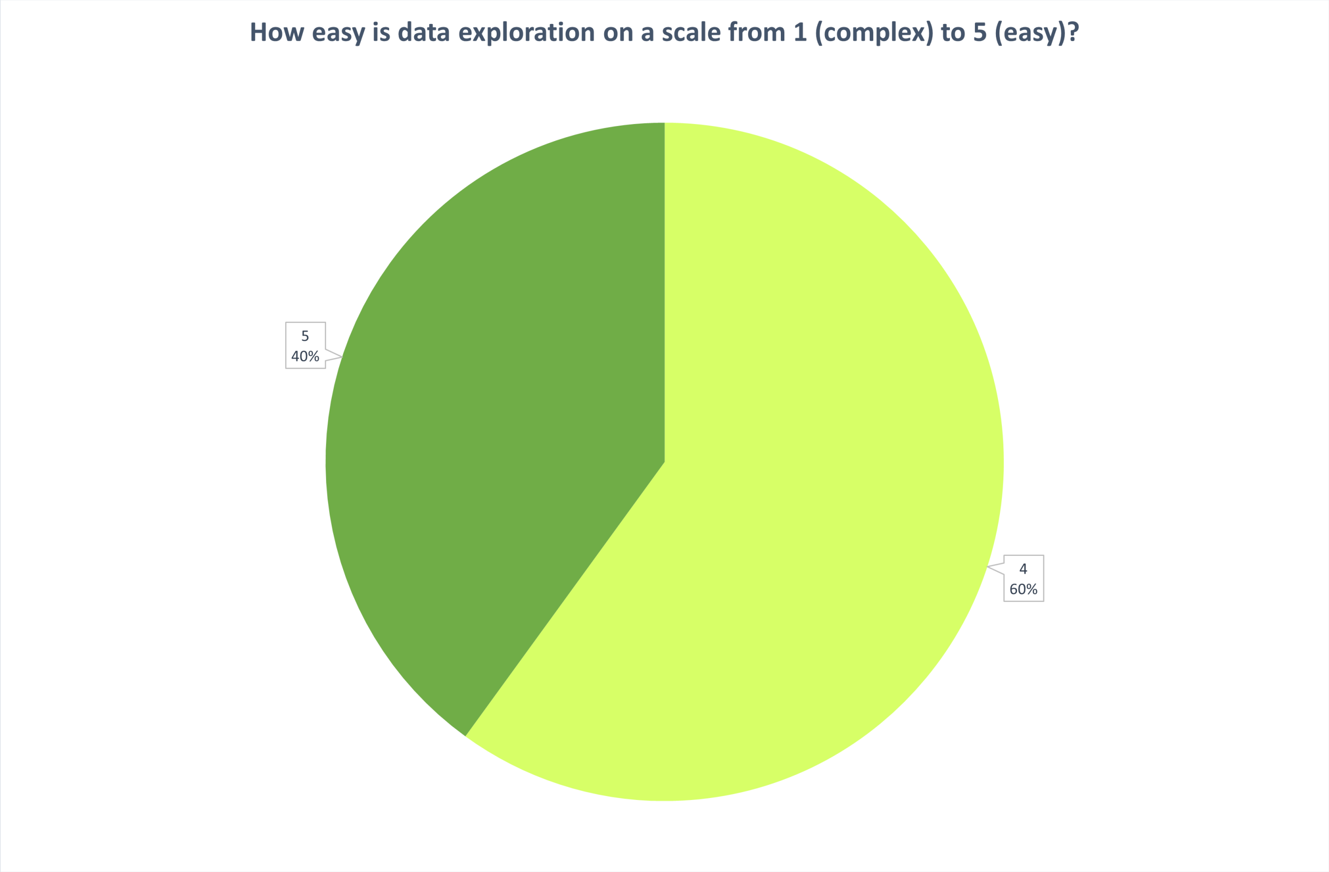

Figure S5.2. distribution of the answers to the question "How easy is data exploration on a scale from 1 (complex) to 5 (easy)?".

S5.2 Question 2

Question 2 was formulated to understand how data entry was perceived in terms of input speed (Figure S5.3). A part of users gave a rather low vote, scoring an average of 3.4 ± 1.6 over 5. The suspect is that data entry is a heavier activity with respect to data exploration, and users may have weighted this aspect. Controls are standard HTML inputs and little can be done to ease the task. Perhaps the procedure could be sped up by reducing the truly useful fields and by collecting more essential data.

S5.3 Question 3

Question 3 is about the user interface, and in this case, the lower vote is higher than in the previous case: 4.2 ± 0.8 (Figure S5.4). The question was intentionally generic, in order to invite all users to express an opinion, but the feeling is that the "intuitiveness" is based on the organization of the menu commands. It is true that there is an easy guide on the main page and that it can be quickly customized, however, MediaWiki forms based on the concept of unique page titles have been replaced by progressive IDs, which are more suited for anonymizing patients' pages, if necessary. This will be worth considering for an investigation, possibly by instrumenting the graphical interface and timing the users' mouse actions.

S5.4 Question 4

Question 4 is about plots and statistical analyses proposed as examples for the data provided by the users (Figure S5.5). As clearly stated, they do not give definitive answers to research questions but are either a way to obtain an overall sense of the stored data or the starting point for further more informative analyses developed by bio-statisticians. The answers have a good average score of 4.3 ± 0.5, however, they present a slice of neutral opinions. This could be due to the fact that the final users are medical doctors that have less familiarity with statistical tests and p-values. In any case, the expressed votes tend to be quite high, and this could mean that there is an awareness of the importance of these scientific tools in the everyday practice of medicine. Perhaps the audience of such a question should be the bio-statisticians supporting the research groups, even if in this scenario they will provide directly the statistical applications and they would be less interested in the particular example applications proposed than in the development of them.

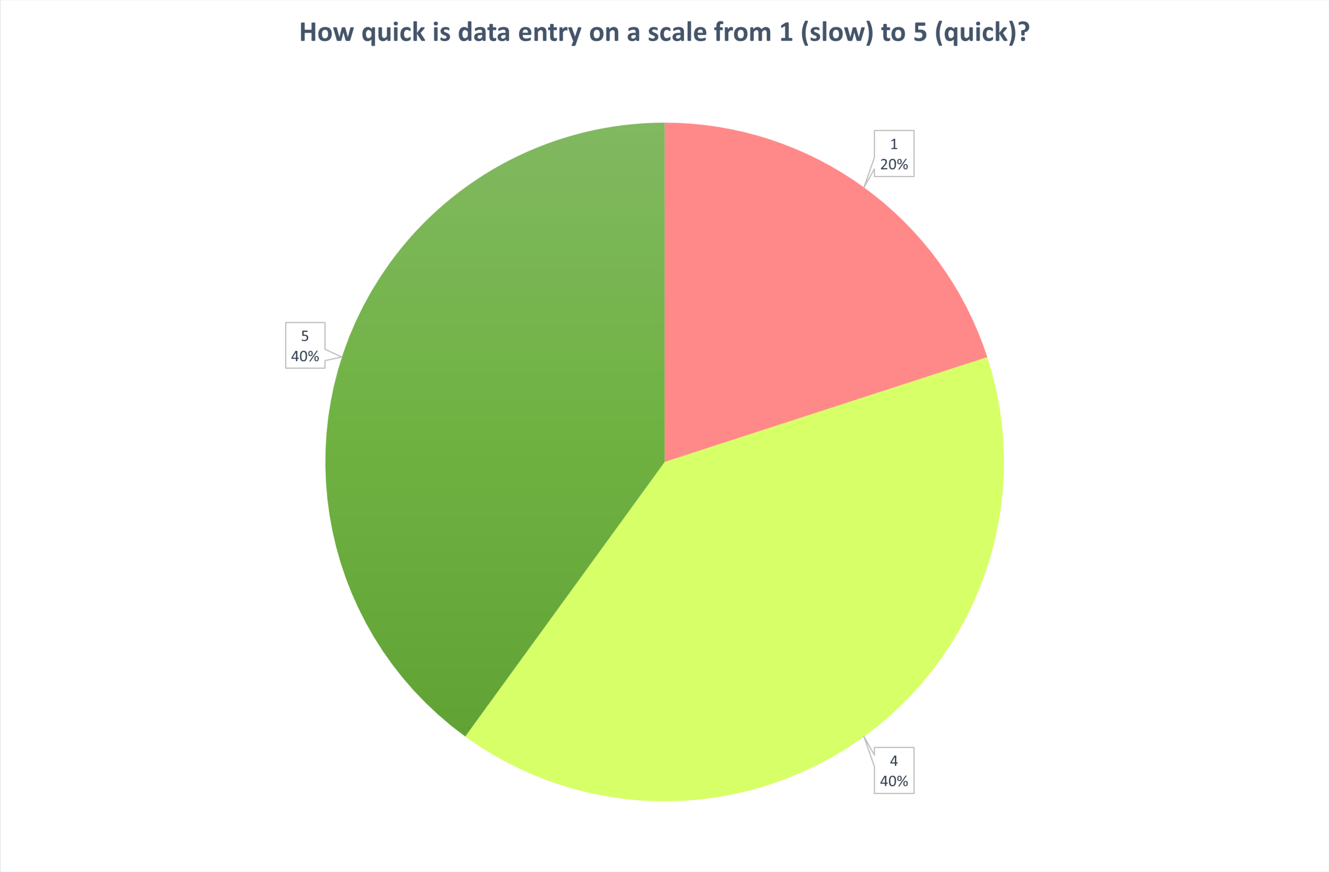

Figure S5.3. distribution of the answers to the question "How quick is data entry on a scale from 1 (slow) to 5 (quick)?".

S5.5 Question 5

The last question obtained the lowest number of answers and scored an average of 4.7 ± 0.6 (Figure S5.6). This is easy to see: it is about the data export, a rather distant step in the process of data collection. It is done when an appropriate number of samples is already present and consistent. Moreover, it is again an operation more typical of a bio-statistician than of a common (medical doctor) user. Another cause of uncertain behavior could be the presence of the Prolog format, which can be appreciated only by a Logician or a Knowledge Engineer. Nonetheless, this is another point that could be addressed, in the sense that the exported documents could be enriched with appropriate formatting and made more similar to familiar formats like for example the ubiquitous Excel format.

System validation among users demonstrates the usability of the developed semantic media wiki interface by bio-medical users and it suggests improvements for the future development of the interface.

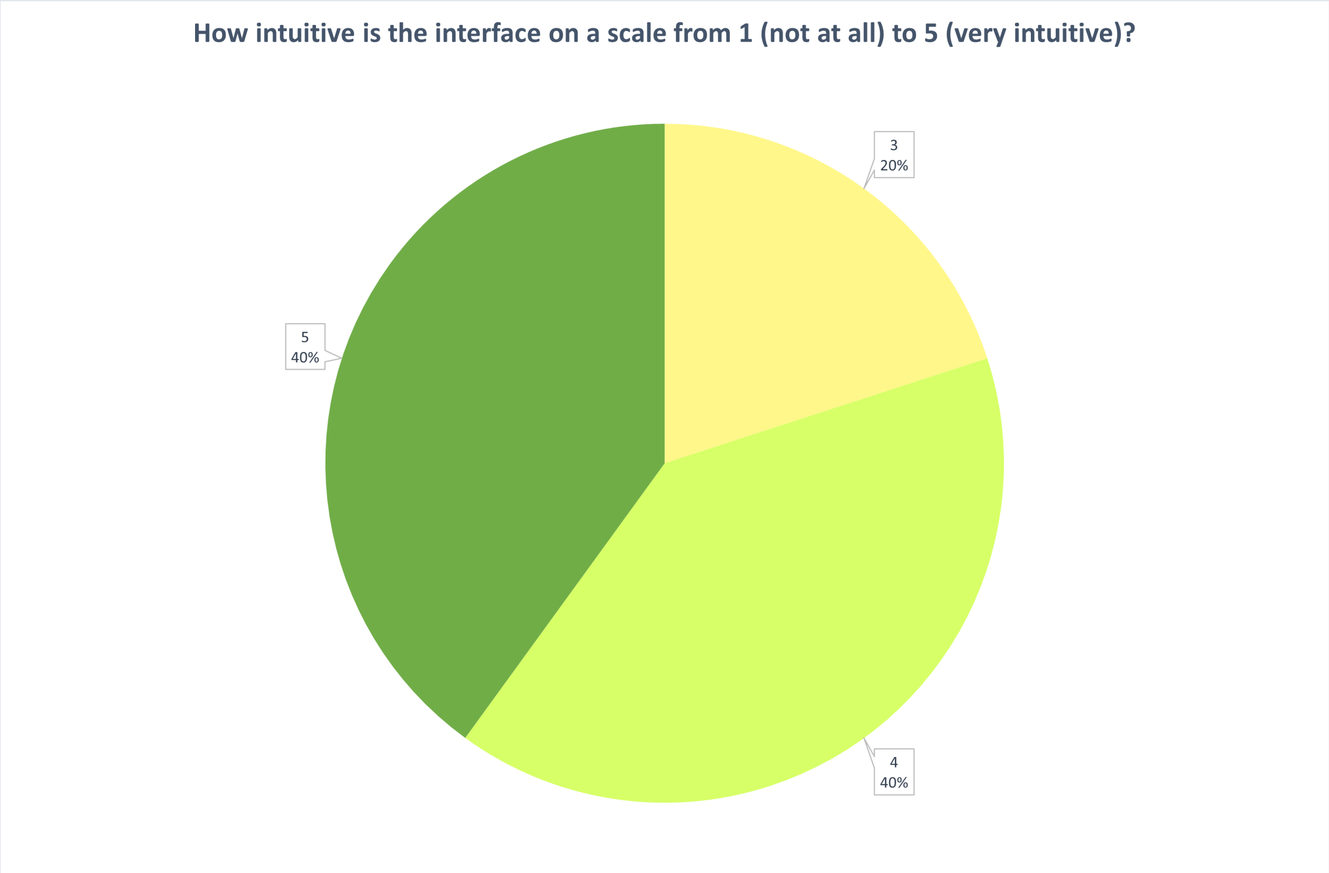

Figure S5.4. distribution of the answers to the question “How intuitive is the interface on a scale from 1 (not at all) to 5 (very intuitive)?”.

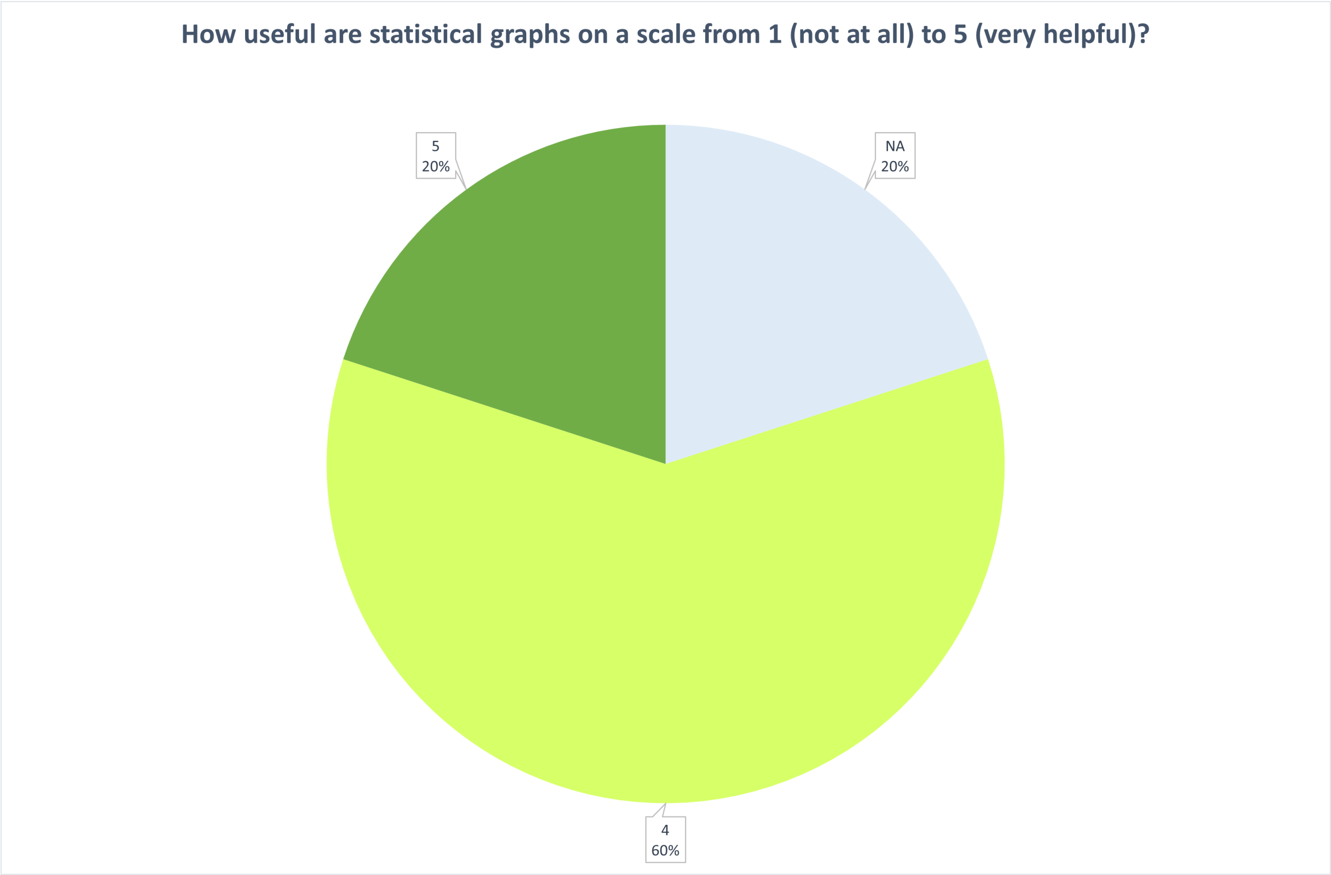

Figure S5.5. distribution of the answers to the question “How useful are statistical graphs on a scale from 1 (not at all) to 5 (very helpful)?”.

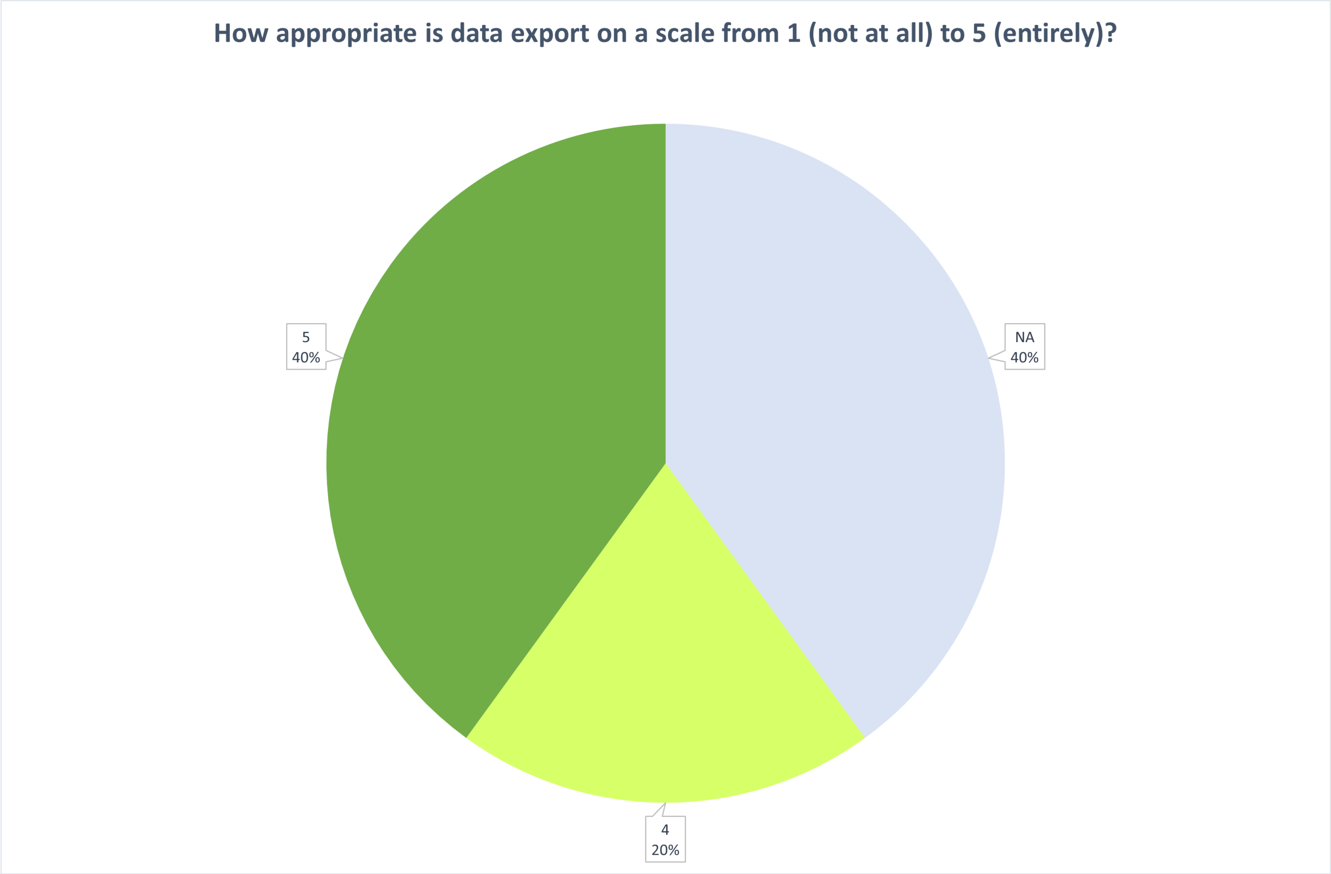

Figure S5.6. distribution of the answers to the question "How appropriate is data export on a scale from 1 (not at all) to 5 (entirely)?".
